# Estimating the population mean for a vertical profile of energy dissipation rate

**DOI:** 10.1038/s41598-020-77414-2

**Published:** 2020-11-23

**Authors:** Nozomi Sugiura, Shinya Kouketsu, Shuhei Masuda, Satoshi Osafune, Ichiro Yasuda

**Affiliations:** 1 Research and Development Center for Global Change, Japan Agency for Marine-Earth Science and Technology, Yokosuka, Japan; 2 Atmosphere and Ocean Research Institute, University of Tokyo, Chiba, Japan

**Keywords:** Physical oceanography, Nonlinear phenomena, Fluid dynamics

## Abstract

Energy dissipation rates are an important characteristic of turbulence; however, their magnitude in observational profiles can be incorrectly determined owing to their irregular appearance during vertical evolution. By analysing the data obtained from oceanic turbulence measurements, we demonstrate that the vertical sequences of energy dissipation rates exhibit a scaling property. Utilising this property, we propose a method to estimate the population mean for a profile. For scaling in the observed profiles, we demonstrate that our data exhibit a statistical property consistent with that exhibited by the universal multifractal model. Meanwhile, the population mean and its uncertainty can be estimated by inverting the probability distribution obtained by Monte Carlo simulations of a cascade model; to this end, observational constraints from several moments are imposed over each vertical sequence. This approach enables us to determine, to some extent, whether a profile shows an occasionally large mean or whether the population mean itself is large. Thus, it will contribute to the refinement of the regional estimation of the ocean energy budget, where only a small amount of turbulence observation data is available.

## Introduction

Numerous existing studies have highlighted the importance of determining energy dissipation rates to investigate ocean general circulation^1,2^. Therefore, several observational studies have been conducted to obtain the vertical profiles of energy dissipation rates using ocean microstructure profilers^3,4^. In addition, to understand the statistics of the irregular evolution of observational profiles, studies have been conducted from the perspective of statistical fluid mechanics, as summarised below.

In fully developed turbulence, an inertial subrange of length scales exists wherein the advective term dominates the molecular viscosity term in the Navier–Stokes equation^5^. In this inertial subrange, a cascade of energies can be observed from large to small scales, as intuitively stated by Richardson^6^. In the first quantitative theory on energy cascades, Kolmogorov^7^ established a relationship wherein velocity fluctuations are locally isotropic and are determined by the homogeneous energy dissipation rate; here, homogeneous means that the statistical property is independent of the position *x*,

1 $$\begin{aligned} \langle |v(x+\ell )-v(x)| \rangle&\approx \varepsilon ^{1/3}\ell ^{1/3}, \end{aligned}$$

where *v* denotes the velocity; $\varepsilon$, the energy dissipation rate; $\ell$, the distance between the points; and $\langle \cdot \rangle$, the expected value. Subsequently, the energy dissipation rate was argued to vary, exhibiting considerable random fluctuations^8^. Thus, a refined theory^9^ was proposed to address this issue. This theory stated that (1) $\log {\varepsilon _r}$, which is the logarithm of the spatially averaged energy dissipation rate over scale *r*, obeys a Gaussian distribution, and (2) its variance obeys $\sigma ^2_{\log {\varepsilon _r}} =A+\mu \log {(L/r)}$, where *L* denotes the outer scale; *A*, a constant associated with the macrostructure of flow; and $\mu$, the intermittency constant.

In addition, several experimental studies^10,11^ demonstrated that small-scale dissipation is a random field that has a spatial structure with power–law correlations,

2 $$\begin{aligned} \langle \varepsilon (x)\varepsilon (x+\ell )\rangle&\propto \ell ^{-\mu },\quad \ell >0. \end{aligned}$$

Then, Yaglom^12^ formulated a quantitative model, which was consistent with the log-normal scaling presented by Kolmogorov^9^ and the power–law correlations, as a multiplicative cascade, where $\varepsilon _{r}$ was expressed with a binary tree comprising independent and identically distributed (i.i.d) random variables, $W_{n',k}$ ($\sim W$),

3 $$\begin{aligned} \forall ~ 1 \le j \le 2^n, \quad \varepsilon _r(x_j)&= \prod _{n'=1}^{n} W_{n',\lfloor (j-1)/2^{n-n'} \rfloor +1}, \end{aligned}$$

where $x_j$ are the positions with equal spacing and $\lfloor s \rfloor$ is the floor function, which assigns the integer that satisfies $0 \le s-\lfloor s \rfloor <1$. If the random variables are set to have the moment exponent $K(q)=\log _{2}\langle W^q\rangle =(\mu /2)(q^2-q)$, then the energy conservation in a probabilistic sense, $\langle W \rangle =1$, and the log-normal scaling in Kolmogorov^9^ are reproduced. Moreover, correlation (2) is reproduced because we have $\langle \varepsilon (x)\varepsilon (x+\ell )\rangle = \langle W^2 \rangle ^{n-m} \langle W \rangle ^{2m} \propto \ell ^{-K(2)}$, where $L=2^n r,~\ell = 2^m r$ for small *r*^12,13^.

Several alternative multiplicative cascade models have been developed with different generators, including the $\beta$ model^14^, random $\beta$ model^15^, $\alpha$ model^16^, *p* model^17^, log-stable model^18^, and log-Poisson model^19^. An important observation regarding Yaglom’s cascade is that the property required for the law of random variable *W* can be formulated such that the product of several random variables still obeys the same class of distribution, $\prod _{n'=1}^n W_{n'} \sim a_n W^{b_n}$, with $a_n,b_n>0$^20^. Consistent with this condition, the universal multifractal model^18^ employs a stable Lévy generator, $\Gamma$, that is maximally left skewed and satisfies $W=\mathrm {e}^{\Gamma }$. This results in a simple and nonanalytic form of the moment exponent, $K(q)=\left( C_1/(\alpha -1)\right) \left( q^{\alpha }-q\right) ,$ where $\alpha$ is the multifractal index, which can be a non-integer, and $C_1$ is the codimension of the mean. The universal multifractal model is the most promising model. This model can well reproduce the variability in several phenomena including turbulence, other geophysical phenomena, and several fractal-like appearances in natural and man-made objects.

Based on this theory, we discuss a refined statistical treatment of the vertical profiles of the observed energy dissipation rates. We first distinguish the ’mean energy dissipation rate’, which refers to the sample (arithmetic) mean over a profile, and the ’energy input rate’, which refers to the population mean for a profile.

Thus, we reconsider one of the basic questions in the observational study of ocean turbulence: When a vertical profile of the energy dissipation rate is given, how can one estimate the energy input rate or the population mean of the energy dissipation rate for a profile, which has been commonly equated with the arithmetic mean over the profile? Our question pertains to whether one can obtain information regarding the energy input rate beyond the arithmetic mean. The answer is yes, because we can construct a model for the turbulent cascade process and solve the inversion problem to obtain the energy input rate under an observational constraint. In this study, we first show that the observed profiles of the depth-averaged energy dissipation rate, $\epsilon _r$, exhibit a scaling property consistent with that of the universal multifractal model. Then, we construct a multiplicative cascade simulation model that describes the statistics of the observational data. Finally, we propose a method to explain certain statistics of the observed profiles based on the simulation model and develop an inversion method to estimate the energy input rate. This result illustrates a systematic method of gaining further quantitative information from profile data.

## Methods

### Observational data

**Figure 1:**
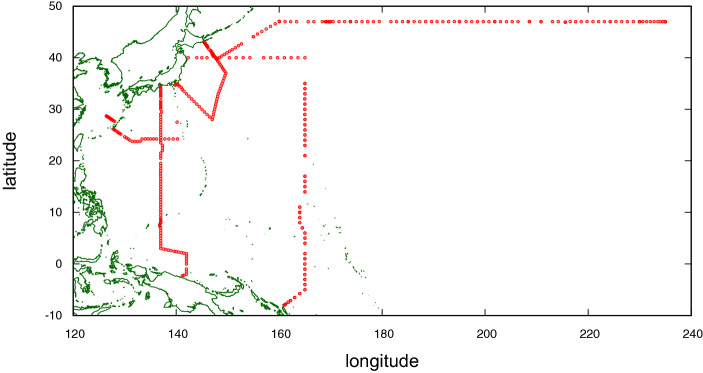
Horizontal locations of the observed profiles (red) and land–sea boundaries (green). The units of longitude and latitude are $^{\circ }~\mathrm {E}$ and $^{\circ }~\mathrm {N}$, respectively.

In this section, we describe the turbulence observational data employed in this study. The data were retrieved from the Pacific Ocean (Fig. 1)^21^. They comprise $I=409$ profiles, each of which typically extends over a depth of 2000–6000 m below the sea surface, in turn comprising observational bins with width of $r_0\simeq 10~\mathrm{m}$. The turbulent energy dissipation rate for each bin, $\epsilon _{r_0}$, is derived by averaging the observational values in the bin, which are estimated from the observed spectrum of the temperature vertical gradient based on the procedure presented in Goto et al.^21,22^ (see Supplementary Information A for the estimation procedure). We restrict our investigation to the intermittency occurring at larger scales, $r \ge r_0$.

**Figure 2:**
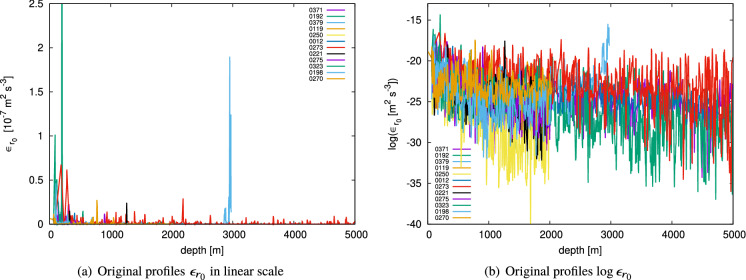
Appearances of observed profiles.

Let $r_0$ be the bin width, $\vec {x}_i$ the horizontal coordinate of the *i*-th profile, and $z^i_j$ the vertical coordinate of the *j*-th point in the *i*-th profile. These positive-valued data exhibit the following characteristics:

1. Each profile defines an ordered set,
  4 $$\begin{aligned}&\left\{ \epsilon _{r_0}(\vec {x}_i,z^i_j)\bigg | j=1,2,\ldots ,J_i\right\} , \end{aligned}$$
  which exhibits an extremely irregular evolution that impedes the recognition of a continuous curve along the depth direction (Fig. 2(a)).
2. After taking the logarithm of the values, the sequences appear to be more continuous (Fig. 2(b)).
3. If we normalise each value with the arithmetic mean along the profile to which it belongs as follows:
  5 $$\begin{aligned} \varepsilon _{r_0}(\vec {x}_i,z^i_j)= \frac{\epsilon _{r_0}(\vec {x}_i,z^i_j)}{\epsilon _L(\vec {x}_i)},~ \epsilon _L(\vec {x}_i) {\mathop {=}\limits ^{\text {def}}}J_i^{-1}\sum _{j=1}^{J_i}\epsilon _{r_0}(\vec {x}_i,z^i_j). \end{aligned}$$
  then the histogram of the logarithmic values,
  6 $$\begin{aligned} \left\{ \log {\left( \varepsilon _{r_0}(\vec {x}_i,z^i_j) \right) } \bigg | i=1,2,\ldots ,I; j=1,2,\ldots ,J_i \right\} , \end{aligned}$$
  appears as an asymmetric distribution, as we will see later in the Results section. Note the distinction between the two symbols; $\epsilon _{r_0}(\vec {x}_i,z^i_j)$ for the original energy dissipation rates, and $\varepsilon _{r_0}(\vec {x}_i,z^i_j)$ for the normalised ones.

### Multifractal analysis

We conduct a scaling analysis of the moments to derive the moment scaling exponent within the universal multifractal framework. Although the analysis could be extended to multidimensional objects^23^, the limited number of samples (409 profiles) prevents us from conducting an extensive analysis in a multidimensional framework. Therefore, we treat each profile as an independent sample and analyse the statistical structure of the 1-dimensional object.

#### Universal multifractal model

The basic formulation of the universal multifractal model is as follows^23,24^: Suppose we have a multifractal field, $\varepsilon _{\lambda }$, at resolution $\lambda$ ($=L/r$), where *r* is the observational scale and *L* is the outer scale. The field is normalised by the mean, that is $\langle \varepsilon _{\lambda }\rangle =\langle \varepsilon \rangle =1$, which is conserved at all scales.

The probability of exceeding a scale-dependent threshold, $\lambda ^{\gamma }$, varies according to singularity $\gamma$ as

7 $$\begin{aligned} \Pr (\varepsilon _{\lambda }\ge \lambda ^{\gamma })&\approx \lambda ^{-c(\gamma )}, \end{aligned}$$

where $c(\gamma )$ represents the codimension function and $\approx$ represents equality up to multiplication by a slowly varying function of $\gamma$. Thus, the multifractal model is characterised by the property that the codimension varies with the singularity. This relation is equivalently represented as the scaling of the statistical moment of any order, *q*,

8 $$\begin{aligned} \langle (\varepsilon _{\lambda })^q\rangle&= \lambda ^{K(q)}, \end{aligned}$$

where *K*(*q*) is the moment scaling function. The two functions, *K*(*q*) and $c(\gamma )$, are actually related by the Legendre transformation because the moment generation function can be written in terms of the occurrence probability of singular events using the saddle-point approximation, $\langle \left( \varepsilon _{\lambda }\right) ^q\rangle =\int \lambda ^{q\gamma }\mathrm {d}p(\gamma )\approx \lambda ^{\max _{\gamma }\left\{ q\gamma -c(\gamma )\right\} }$^25^, where $\mathrm {d}p(\gamma ){\mathop {=}\limits ^{\text {def}}}\Pr (\lambda ^{\gamma } \le \varepsilon _{\lambda }< \lambda ^{\gamma +\mathrm {d}\gamma })$. Functions *K*(*q*) and $c(\gamma )$ determine the variability of the multifractal field $\varepsilon _{\lambda }$ across the scales, $\lambda$.

Owing to a generalisation of the central limit theorem, several multiplicative processes comprising different generators converge to a universal multifractal^18,26^, the moment exponent of which is expressed as follows:

9 $$\begin{aligned} K(q)&=\frac{C_1}{\alpha -1}(q^{\alpha }-q), \end{aligned}$$

where $0\le \alpha \le 2$ is the multifractal index and $C_1$ is the codimension of the mean. Note that the case $\alpha = 2$ corresponds to the log-normal model advocated by the Russian school (Kolmogorov, Obukhov, Yaglom, etc.). This equation satisfies probability normalisation, $K(0)=0$, and energy conservation, $K(1)=0$. Its Legendre transformation gives:

10 $$\begin{aligned} c(\gamma )&= C_1\left( \frac{\gamma }{C_1 \alpha '}+\frac{1}{\alpha }\right) ^{\alpha '}, \end{aligned}$$

where $$1/\alpha +1/\alpha '=1$$.

### Estimations based on the cascade model

In this section, we discuss the estimation of the energy input rate, ${\overline{\epsilon }}$, by utilising the information obtained from an observational profile. While the sample mean of the energy dissipation rate along a profile is simply indicated by the arithmetic mean of the vertical data values, the information on the energy input rate and its uncertainty still needs to be obtained. Therefore, we will estimate the posterior distribution of the energy input rate from observations. In particular, we focus on the median and confidence interval (CI). Although the arithmetic mean over a profile is the primary measure for the sample, the characteristics of the population can also be evaluated by using the joint probability density of several different sample statistics, obtained from the Monte Carlo simulation of the cascade model. The notation used in this section is summarised in Table 1.

**Table 1:** Notation for the estimation study.

Name	Notation	Definition
Index for vertical position	*j*	$1,2,3,\ldots , 2^n$
Energy dissipation rate	$\epsilon _j$	
Logarithm of energy dissipation rate	$\gamma _j$	$\log {\epsilon _j}$
Energy input rate (or population mean)	${\overline{\epsilon }}$	
Logarithm of energy input rate	${\overline{\gamma }}$	$\log {{\overline{\epsilon }}}$
Median of estimated ${\overline{\gamma }}$	$\gamma _{(0.5)}$	$\Pr ({\overline{\gamma }}<\gamma _{(0.5)})=0.5$
Stable Lévy generators	$\Gamma _{ik}$	$\sim S_{\alpha }(\sigma h^{1/\alpha },-1,-\widehat{\sigma _{\alpha }}^{\alpha }h)$
Width of Lévy generator	–	$\sigma h^{1/\alpha }$
Shift of Lévy generator	–	$-\widehat{\sigma _{\alpha }}^{\alpha }h$ = $-\frac{\sigma ^{\alpha }}{\cos {\left( \frac{\pi }{2}(2-\alpha )\right) }} h =-\frac{C_1}{\alpha -1} h$
Logarithm of arithmetic mean	${\widehat{\gamma }}$	$\log {\left( 2^{-n}\sum _{j=1}^{2^n}\mathrm {e}^{\gamma _j}\right) }$
Logarithm of geometric mean	${\widetilde{\gamma }}$	$2^{-n}\sum _{j=1}^{2^n}\gamma _j$
Logarithm of quadratic mean	$\gamma ^{\sharp }$	$2^{-1}\log {\left( 2^{-n}\sum _{j=1}^{2^n} \mathrm {e}^{2\gamma _j}\right) }$
Marginal probability density function	$q_1$	Probability density of ${\overline{\gamma }}-{\widehat{\gamma }}$;
		$$q_1(\cdot )=\int \int q_3(\cdot ,u,v)\mathrm {d}u \mathrm {d}v $$
Joint probability density function	$q_3$	Probability density of $({\overline{\gamma }}-{\widehat{\gamma }}, {\widehat{\gamma }}-{\widetilde{\gamma }}, {\widehat{\gamma }}-\gamma ^{\sharp })$

#### Multiplicative cascade simulation

**Figure 3:**
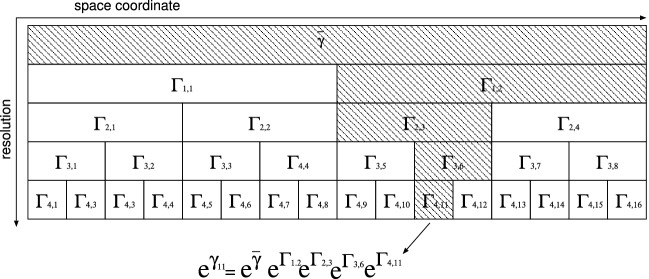
Schematic of the multiplicative cascade model. The energy dissipation rate at $z_{11}$ at resolution $r_4=L/2^4$ is considered as an example.

 To examine the relationship between various statistical quantities derived from observational profiles, we construct a simulation model for the multiplicative cascade by following the procedure described in Schmitt^27^, as shown in Fig. 3. Each building block, $\Gamma _{ik}$, is a generator that obeys a left-skewed stable distribution, $S_{\alpha }(\sigma h^{1/\alpha },-1,-\widehat{\sigma _{\alpha }}^{\alpha }h)$, with $h=\log {2},~ \widehat{\sigma _{\alpha }}^{\alpha }{\mathop {=}\limits ^{\text {def}}}\sigma ^{\alpha } /\cos {\left( \frac{\pi }{2}(2-\alpha )\right) } =C_1/(\alpha -1)$^28^.

Consider a fixed horizontal position $\vec{x}$. Let ${\overline{\epsilon }}=\exp {\left( {\overline{\gamma }}\right) }$ be the energy input rate for a profile at $\vec{x}$, $n=\log _{2}\frac{L}{r}$ be the number of steps, $0 \le n' \le n$ be the scale index, and $1 \le j \le 2^n$ be the spatial index. The cascade simulation is performed for variable $X_{n',j}$ as follows.

1. For each spatial index $j=1,2,\ldots ,2^n$, set $X_{0,j}={\overline{\gamma }}$.
2. For each scale index $n'=1,\ldots ,n$, repeat the following steps:
  For each spatial block $k=1,2,\ldots ,2^{n'}$, perform the following steps:
       (a)  Generate a random variable, $\xi _{n'k}$, which obeys $S_{\alpha }(1,-1,0)$^29^.
      (b)  For each spatial index $j=(k-1)\cdot 2^{n-n'}+1,\ldots ,k\cdot 2^{n-n'}$, downscale *X* by
    11 $$\begin{aligned} X_{n',j}&= X_{n'-1,j}+\Gamma _{n',k},\quad \Gamma _{n',k}~{\mathop {=}\limits ^{\text {def}}}~-\widehat{\sigma _{\alpha }}^{\alpha } h + \sigma h^{\frac{1}{\alpha }} \xi _{n'k}. \end{aligned}$$
  For each spatial index $j=1,2,\ldots ,2^n$, set $\gamma _j=X_{n,j}$.

The output, $\gamma _j$, represents the logarithm of the energy dissipation rate at the horizontal position, $\vec{x}$, and the vertical position, $z_j \in [(j-1) r_n, j r_n]$, at the resolution $r_n=L/2^n$. By using the floor function, the cascade process can be more compactly represented as

12 $$\begin{aligned} \gamma _j= {\overline{\gamma }} +\sum _{n'=1}^{n} \Gamma _{n',\lfloor (j-1)/2^{n-n'} \rfloor +1}, \quad j=1,2,\ldots ,2^n. \end{aligned}$$

An important implication of this formulation is that the arithmetic mean of the vertical data points is not necessarily equal to the the energy input rate because the cascade process has a fluctuating nature. In other words, a realisation of the vertical average, $\epsilon _L =2^{-n}\sum _{j=1}^{2^n} \exp {\left( \gamma _j\right) }$, is not always equal to $\exp {({\overline{\gamma }}})$, whereas the expectation ${{\,\mathrm{\mathbb {E}}\,}}\left[ \epsilon _L\right]$ is; hence, we can regard the latter as the population mean for a profile. Below, we focus mainly on the relationship between the arithmetic mean over a profile and the energy input rate. We perform statistical estimations from one to the other of these quantities based on the cascade model.

#### Estimation of the energy input rate

In this subsection, we first describe the statistical relationship between the population mean and various moments over a profile, based on the cascade model. Then, we derive a formula for the posterior probability given the observation of the moments. Finally, we use this formula as the basis of a concrete procedure for computing the posterior probability.

We assume that each set of $\gamma _j$’s is generated by an *n*-step cascade model as in Eq. (12). Here, we want to estimate the energy input rate ${\overline{\gamma }}$, which corresponds to the population mean, by using the information from the observed data $\{\gamma _j|j=1,2,\ldots ,2^n\}$. In this regard, in addition to the arithmetic mean ${\widehat{\gamma }}$, which corresponds to *K*(1) in Fig. 5, we can also use other moments over a profile, e.g., the geometric mean ${\widetilde{\gamma }}$ and quadratic mean $\gamma ^{\sharp }$, which correspond to $K'(0)$ and *K*(2), respectively. We can derive the following expressions based on Eq. (12):

13 $$\begin{aligned} {\widehat{\gamma }}&={\overline{\gamma }} +\log {\left\{ 2^{-n}\sum _{j=1}^{2^n}\exp {\left( \sum _{n'=1}^{n}\Gamma _{n', \lfloor (j-1)/2^{n-n'} \rfloor +1}\right) }\right\} }, \end{aligned}$$

14 $$\begin{aligned} {\widetilde{\gamma }}&={\overline{\gamma }} + 2^{-n}\sum _{j=1}^{2^n}\sum _{n'=1}^{n}\Gamma _{n',\lfloor (j-1)/2^{n-n'} \rfloor +1}, \end{aligned}$$

15 $$\begin{aligned} \gamma ^{\sharp }&={\overline{\gamma }} +\frac{1}{2}\log {\left\{ 2^{-n}\sum _{j=1}^{2^n}\exp {\left( 2\sum _{n'=1}^{n}\Gamma _{n',\lfloor (j-1)/2^{n-n'} \rfloor +1}\right) }\right\} }, \end{aligned}$$

where we find that the term ${\overline{\gamma }}$ is factored out. Therefore, ${\overline{\gamma }}-{\widehat{\gamma }}$, ${\widehat{\gamma }}-{\widetilde{\gamma }}$, and ${\widehat{\gamma }}-\gamma ^{\sharp }$ are independent of ${\overline{\gamma }}$, and thus dimensionless.

The structure of the cascade model implies that the appearance probability of ${\widehat{\gamma }}$ given ${\overline{\gamma }}$ is determined only by their difference: $P({\widehat{\gamma }}|{\overline{\gamma }}) =q_1({\overline{\gamma }}-{\widehat{\gamma }})$. Furthermore, by assuming that we have no prior information about ${\overline{\gamma }}$, Bayes’ theorem is applied to invert it into the posterior probability for ${\overline{\gamma }}$ as follows.

16 $$\begin{aligned} P({\overline{\gamma }}|{\widehat{\gamma }})&= \frac{P({\widehat{\gamma }}|{\overline{\gamma }})P({\overline{\gamma }})}{\int P({\widehat{\gamma }}|{\overline{\gamma }})P({\overline{\gamma }}) \mathrm {d}{\overline{\gamma }}} = q_1({\overline{\gamma }}-{\widehat{\gamma }}). \end{aligned}$$

Furthermore, we can extract information from ${\widetilde{\gamma }}$ and $\gamma ^{\sharp }$. They are encoded in the joint probability density function (PDF) $q_3({\overline{\gamma }}-{\widehat{\gamma }},{\widehat{\gamma }}-{\widetilde{\gamma }}, {\widehat{\gamma }}-\gamma ^{\sharp })$ computed from Monte Carlo simulation of the cascade model. Then, a conditional PDF is derived as

17 $$\begin{aligned} q_3({\overline{\gamma }}-{\widehat{\gamma }}|{\widehat{\gamma }}-{\widetilde{\gamma }}, {\widehat{\gamma }}-\gamma ^{\sharp })&= \frac{ q_3({\overline{\gamma }}-{\widehat{\gamma }}, {\widehat{\gamma }}-{\widetilde{\gamma }}, {\widehat{\gamma }}-\gamma ^{\sharp } ) }{ \int q_3({\overline{\gamma }}-{\widehat{\gamma }},{\widehat{\gamma }}-{\widetilde{\gamma }}, {\widehat{\gamma }}-\gamma ^{\sharp }) \mathrm {d}{\overline{\gamma }} }. \end{aligned}$$

A procedure similar to Eq. (16) can be applied to obtain another posterior probability:

18 $$\begin{aligned} P({\overline{\gamma }}|{\widehat{\gamma }}, {\widehat{\gamma }}-{\widetilde{\gamma }}=u, {\widehat{\gamma }}-\gamma ^{\sharp }=v)&= q_3({\overline{\gamma }}-{\widehat{\gamma }}|u,v), \end{aligned}$$

under the constraints ${\widehat{\gamma }}-{\widetilde{\gamma }}=u, {\widehat{\gamma }}-\gamma ^{\sharp }=v$.

On the basis of the above formulation, we perform an identical twin experiment obeying the following procedure.

1. Perform a Monte Carlo experiment to obtain $q_3({\overline{\gamma }}-{\widehat{\gamma }}, {\widehat{\gamma }}-{\widetilde{\gamma }}, {\widehat{\gamma }}-\gamma ^{\sharp })$.
  - Set the energy input rate to ${\overline{\gamma }}=0$.
  - Create many random samples of the profile using the cascade model in Eq. (12).
  - Add up the frequency of occurrence to derive the joint PDF $q_3({\overline{\gamma }}-{\widehat{\gamma }}, {\widehat{\gamma }}-{\widetilde{\gamma }}, {\widehat{\gamma }}-\gamma ^{\sharp })$.
2. Set the energy input rate ${\overline{\gamma }}$ to a random number.
3. Create a random pseudo-observation sample of the profile using the cascade model in Eq. (12).
4. Calculate the statistics ${\widehat{\gamma }}, {\widehat{\gamma }}-{\widetilde{\gamma }}, {\widehat{\gamma }}-\gamma ^{\sharp }$ for the profile.
5. Compute the conditional PDF $P({\overline{\gamma }}|{\widehat{\gamma }}, {\widehat{\gamma }}-{\widetilde{\gamma }},{\widehat{\gamma }}-\gamma ^{\sharp })$.
6. Calculate the median and $95\%$ CI for the estimated ${\overline{\gamma }}$.
7. Compare the estimate of ${\overline{\gamma }}$ with its true value.

The same procedure is applied to the real data experiment, except that ${\overline{\gamma }}$ in 2 is unknown, as follows.

1. Pick an observed profile, and calculate the statistics ${\widehat{\gamma }}, {\widehat{\gamma }}-{\widetilde{\gamma }}, {\widehat{\gamma }}-\gamma ^{\sharp }$ for the profile.
2. Compute the conditional PDF $P({\overline{\gamma }}|{\widehat{\gamma }}, {\widehat{\gamma }}-{\widetilde{\gamma }},{\widehat{\gamma }}-\gamma ^{\sharp })$, using the joint PDF $q_3({\overline{\gamma }}-{\widehat{\gamma }}, {\widehat{\gamma }}-{\widetilde{\gamma }}, {\widehat{\gamma }}-\gamma ^{\sharp } )$ obtained from the Monte Carlo experiment.
3. Calculate the median and $95\%$ CI for the estimated ${\overline{\gamma }}$.

Among the indices for the estimated result, the conditional expectation of ${\overline{\epsilon }}=\exp {({\overline{\gamma }})}$ is not necessarily defined as a finite value because the posterior distribution of ${\overline{\gamma }}$ is neither Gaussian nor left-skewed stable. In contrast, the percentiles, including the median and the $95\%$ CI, are always defined for the distribution. They are also preserved, $\Pr ({\overline{\gamma }}<a)=\Pr (f({\overline{\gamma }})<f(a))$, under the increasing transformation $f:{\overline{\gamma }} \mapsto \exp {({\overline{\gamma }})}$. We therefore employ the median and the $95\%$ CI as robust indices.

To confirm that the uncertainty in the estimated values of $\alpha$ and $C_1$ does not diminish the performance of the proposed method, we treat these parameters as random variables (specified in the results), when making pseudo-observation samples in the identical twin experiments (procedure 3).

We have thus established a procedure for estimating the population mean for a profile based on the joint probability distribution of several moments over a profile computed by a Monte Carlo simulation of the cascade model.

## Results

### Analysis of observational data

Suppose we have the observational data of the normalised energy dissipation rate, $\varepsilon _{r_0}(\vec {x})$, in bin width $r_0$ at the horizontal position $\vec {x}$, as well as their spatial average $\varepsilon _{r}(\vec {x})$ in width $r \ge r_0$. In terms of the universal multifractal model (8), the scaling of the statistical moments in the data takes the form

19 $$\begin{aligned} \frac{\langle \varepsilon _{r_0}(\vec {x})^q \rangle }{\langle \varepsilon _{r} (\vec {x})^q \rangle }&= \left( \frac{r}{r_0}\right) ^{K(q)}, \end{aligned}$$

where $\langle \cdot \rangle$ denotes the expected value. This implies that the expectation of the *q*-th moment at a scale over the one at another scale should be equal to the *K*(*q*)-th power of the resolution ratio, regardless of the horizontal position $\vec {x}$. We approximate the expected value in Eq. (19) with the empirical average

20 $$\begin{aligned} \langle \varepsilon _{r}(\vec {x})^q\rangle&\fallingdotseq \frac{\sum _{i=1}^I\sum _{k=1}^{J_i(r)} \left( \varepsilon _{r}^{(i,k)}\right) ^q}{\sum _{i=1}^I J_i(r)},\quad \varepsilon _{r}^{(i,k)} {\mathop {=}\limits ^{\text {def}}}2^{-n}\sum _{j=2^n(k-1)+1}^{2^n k}\varepsilon _{r_0}(\vec {x}_i,z^i_j), \end{aligned}$$

where $r=2^n r_0$ is a resolution larger than or equal to $r_0$, and $\varepsilon _{r_0}(\vec {x}_i,z^i_j)$ is the normalised value defined in Eq. (5). The superscript (*i*, *k*) runs across all profiles indexed by *i*, each of which has total $J_i(r)$ segments in resolution *r*. By substituting Eq. (20) into Eq. (19), we can evaluate the values of *K*(*q*) according to *q*.

**Figure 4:**
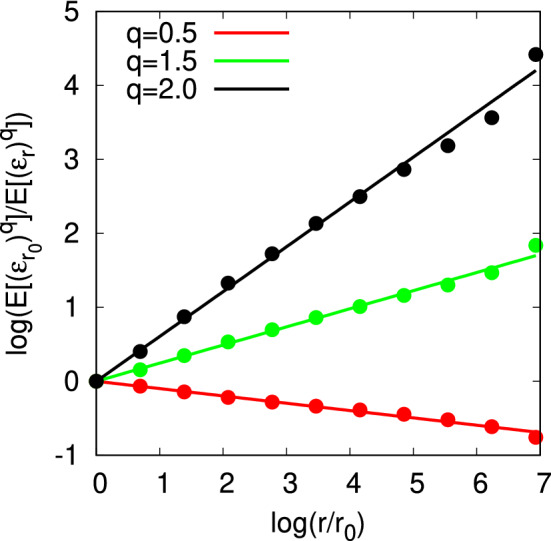
Scale dependency of the moments, $\left( \log {(r/r_0)},-\log {\langle (\varepsilon _r/\varepsilon _{r_0})^{q}\rangle }\right) ,$ where $r_0$ is the width of the observational bin. The moment scaling exponents are found to be $K(0.5)=-0.099\pm 0.003,~K(1.5)=0.245\pm 0.007,~K(2.0)=0.606\pm 0.017$.

**Figure 5:**
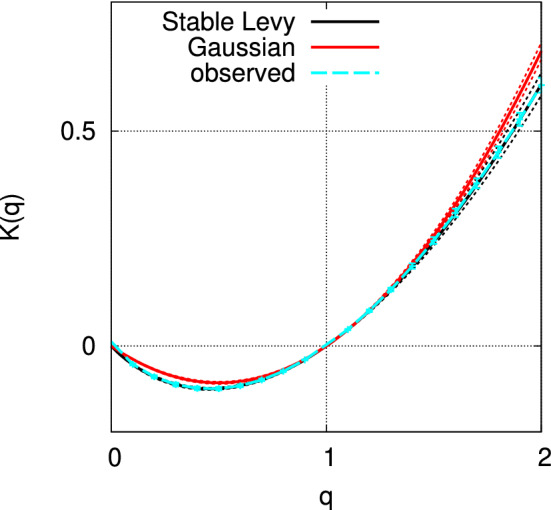
Moment scaling exponent *K*(*q*) for observational data (cyan). Best-fitting multifractal model with stable Lévy generators (black), and with Gaussian generators (red). Each error bar in cyan shows the standard deviation for the fitting of *K*(*q*). Dotted lines in black and red indicate the ranges of error due to the uncertainty of parameters in the corresponding models.

The scalings for several moments are shown in Fig. 4. Using the various slope values, the observational curve of (*q*, *K*(*q*)) in the range of $0 \le q\le 2$ is indicated in Fig. 5 in cyan.

We can estimate the parameters, $\alpha$ and $C_1$, by fitting the theoretical curve (9) to the observational curve. To consider the uncertainty in the observational curve, we used the bootstrap method^30^ with 1000 trials, each of which has 409 profiles that are randomly sampled with replacements from the original set of 409 profiles. We thereby obtained the parameters $\alpha = 1.62 \pm 0.03,\quad C_1 = 0.352 \pm 0.009$ for the multifractal model with stable Lévy generators, i.e., the universal multifractal model. By taking into account the dependency on $\alpha$, we can also estimate $C_1$ as $C_1=0.109\alpha +0.175\pm 0.008$. By a similar procedure, we obtain the parameter $C_1=0.343\pm 0.010$ for the multifractal model with Gaussian generators, corresponding to the original Yaglom cascade with $\mu =2C_1$. In Fig. 5, the observational curve (cyan) and the theoretical curve for the multifractal model with stable Lévy generators (black) are in good agreement, while the theoretical curve for the multifractal model with Gaussian generators, i.e., the log-normal model (red), has a different curvature from the observational curve.

The parameter values for the multifractal model with stable Lévy generators are largely consistent with previous results for atmospheric dissipation fields ($\alpha =1.35\pm 0.07,~C_1=0.3\pm 0.05$ for the horizontal shear of a velocity field^31^; $\alpha =1.85\pm 0.05,~C_1=0.59\pm 0.05$ for vertical kinetic energy flux^32^).

**Figure 6:**
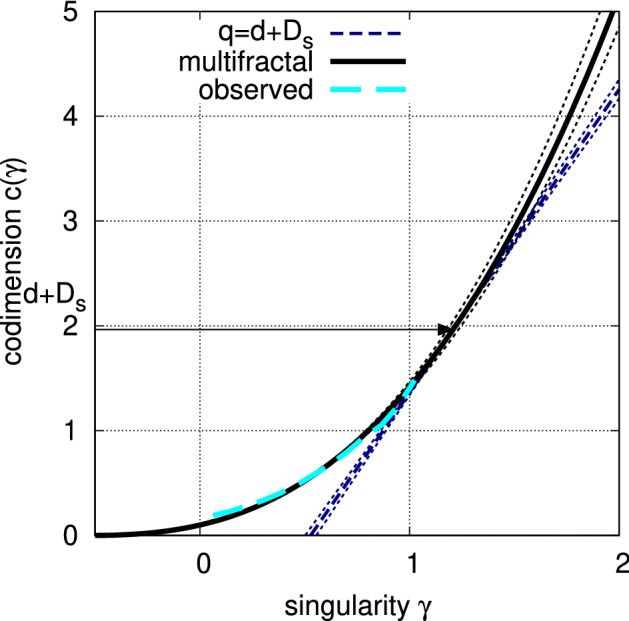
Codimension $c(\gamma )$ of singularities $\gamma$ for the best-fitting multifractal model with stable Lévy generators (black). The corresponding curve for the observational data is shown for reference (cyan). Sampling dimension $D_s$ and the limitation for the moment exponent (the slope of the navy-blue line) are also shown. Dotted lines in black and navy indicate the ranges of error due to the uncertainty in the model parameters.

Figure  6 shows the theoretical curve of extremes for the multifractal model (10) in black and the observational curve,

21 $$\begin{aligned} c_{\text {obs}}(\gamma )&=-\log _{\lambda }\left[ g(\gamma ){\Pr \left( \varepsilon _{r_0}> \lambda ^{\gamma }\right) } \right] ,\quad \lambda =L/r_0, \end{aligned}$$

in cyan, where $\lambda =2^9$ is used; this is a typical scale ratio in the data. Note that the correction term,

22 $$\begin{aligned} g(\gamma )&=\sqrt{2\pi \alpha c(\gamma ) \log {\lambda }}, \end{aligned}$$

compensates for the prefactor in the asymptotic complementary cumulative distribution function, $g(\gamma )^{-1}\mathrm {e}^{-c(\gamma )}$^28^, Eq. 1.2.11]. The two curves (10) and (21) appear to be in general agreement, except for a slight discrepancy that is possibly due to the ambiguity in the selected scale ratio, $\lambda$. Moreover, as our data have the sampling dimension^23^ $D_s=\log _{\lambda }{N_s}\simeq \log {409}/\log {(2^9)}=0.963$, the upper bound for *q* is calculated to be $q_s=2.89\pm 0.11$ (the slope of the navy-blue line in Fig. 6), which justifies the range we set ($0 \le q\le 2$).

**Figure 7:**
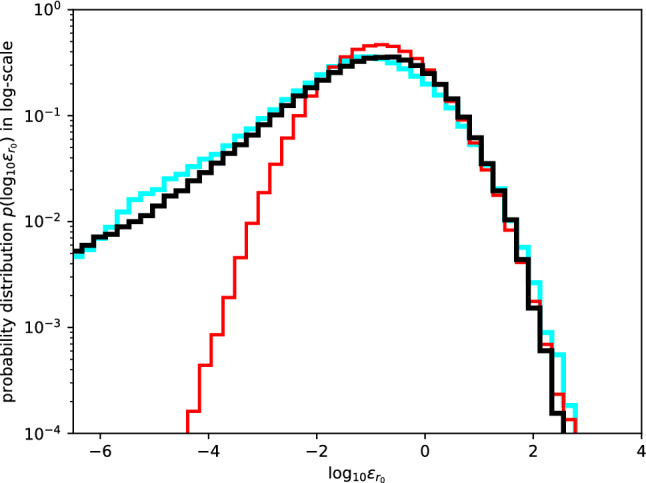
Distribution of the logarithm of observational data normalised for each profile (cyan), and comparison with the statistics of samples generated from multiplicative cascade with Gaussian/stable Lévy generators (red/black).

To demonstrate the appropriateness of the universal multifractal model, the histogram for the logarithm of the bin values in the observational data is shown in Fig. 7 and compared with the samples from multiplicative cascade models. Each bin value is normalised by the arithmetic mean along the profile it belongs to: $\varepsilon _{r_0}=\epsilon _{r_0}/\epsilon _L.$ The histogram for the logarithm of bin data, $\log _{10}{\varepsilon _{r_0}}$, appears to be in good agreement with the histogram of samples generated by the 8-step cascade model with stable Lévy generators (black; $\alpha = 1.62, ~C_1=0.352$) and in poor agreement with that generated by the multifractal model with Gaussian generators, i.e., the log-normal model (red; $\alpha = 2, ~C_1=0.343$).

**Table 2:** Constants and parameters for the estimation study.

Meaning	Parameter	Value
Number of steps in cascade	*n*	8
Number of vertical points	$2^n$	256
Step size of cascade	*h*	$\log {2}$
Number of samples in Monte Carlo simulation	*M*	$1.024\times 10^{10}$
Multifractal index	$\alpha$	1.62
Codimension of the mean	$C_1$	0.352
Width of bins in histogram of $q_3$	$(w_1,w_2,w_3)$	(0.1, 0.1, 0.05)
Number of bins in histogram of $q_3$	$(n_1,n_2,n_3)$	(500, 200, 200)
Number of profiles in identical twin exp.	$M'$	30,000
Number of observed profiles (Obs.)	I	409
Obs. with more than $2^n$ vertical points	–	353

Moreover, in the same manner as the correlation in Yaglom’s cascade, the observational profiles have a power-law autocorrelation,

23 $$\begin{aligned} \langle \varepsilon (z)\varepsilon (z+\ell )\rangle&\propto \ell ^{-K(2)}=\ell ^{-0.609}, ~\ell >0, \end{aligned}$$

where $\varepsilon (z)$ is the energy dissipation rate at depth *z*. The negative exponent explains the discontinuous characteristics observed in the profiles (see Fig. 2a).

### Simulations of cascade model

Before estimating the energy input rate ${\overline{\epsilon }}$ that corresponds to each observational profile, we first performed a Monte Carlo experiment with simulations of $1.024\times 10^{10}$ particles (profiles) using the 8-step cascade model. The constants and parameters used in the simulation and estimation study are summarised in Table 2.

**Figure 8:**
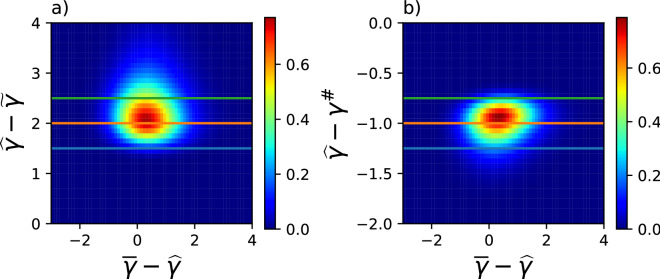
Examples of cross section cut of joint probability density function $q_3({\overline{\gamma }}-{\widehat{\gamma }}, {\widehat{\gamma }}-{\widetilde{\gamma }}, {\widehat{\gamma }}-\gamma ^{\sharp })$. (**a**) section cut $q_3(\cdot ,\cdot ,-1)$ with section lines ${\widehat{\gamma }}-{\widetilde{\gamma }}=1.5, 2, 2.5$, and (**b**) section cut $q_3(\cdot ,2,\cdot )$ with section lines ${\widehat{\gamma }}-\gamma ^{\sharp }=-1.25, -1, -0.75$.

For each particle (or profile), we generate random numbers $\{\gamma _j|j=1,2,\ldots ,256\}$ from ${\overline{\epsilon }}=1$ according to the procedure discussed in Methods, and we add up the histograms for all the particles into the joint PDF $q_3({\overline{\gamma }}-{\widehat{\gamma }}, {\widehat{\gamma }}-{\widetilde{\gamma }}, {\widehat{\gamma }}-\gamma ^{\sharp })$ (Fig. 8).

**Figure 9:**
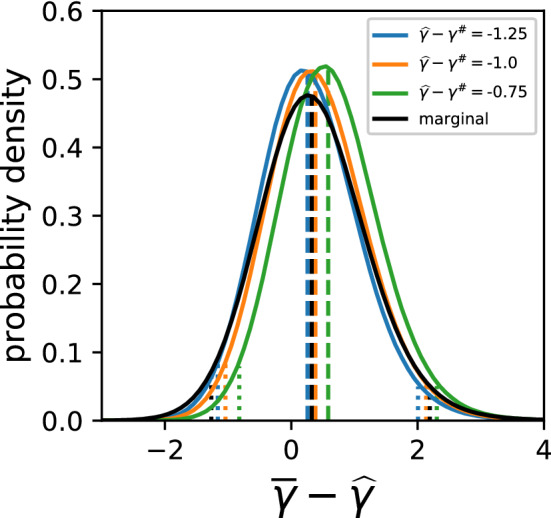
Examples of conditional probability density function $q_3(\cdot |u,v)$ (coloured) and marginal probability distribution $q_1(\cdot )$ (black), along with median (dashed lines) and $95\%$ confidence intervals (dotted lines). The conditional probabilities are for $u=2; v=-1.25, -1, -0.75$.

Examples for conditional PDF $q_3({\overline{\gamma }}-{\widehat{\gamma }}| {\widehat{\gamma }}-{\widetilde{\gamma }}, {\widehat{\gamma }}-\gamma ^{\sharp })$ are shown in Fig. 9.

### Identical twin experiment

Second, we estimated the posterior probability distribution for the energy input rate, ${\overline{\epsilon }}$ by inverting the probability distribution computed from the simulation of the cascade model. Before applying this to real data, we performed an identical twin experiment using pseudo-observational data, whose energy input rates were given manually; thus, the estimation result could be checked against them. The inversion was performed using the result of the joint PDF $q_3$ created by the cascade model with the Lévy generator (case L3). When generating the pseudo-observational data, the parameters for each profile were set randomly as $\alpha = 1.62 + 0.03\xi _1,~ C_1=0.109\alpha +0.175 + 0.008\xi _2,$ using standard normal random numbers, $\xi _1$ and $\xi _2$.

**Figure 10:**
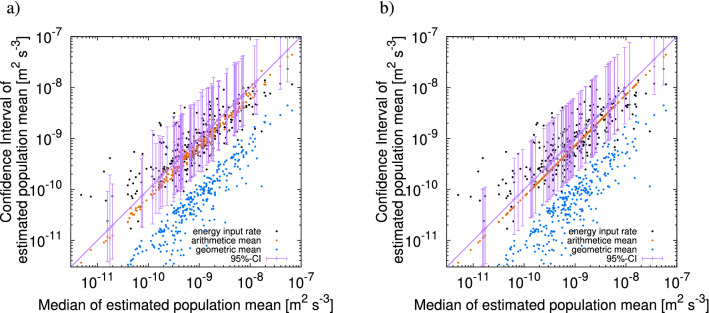
Result of identical twin experiments. Median (horizontal axis) versus confidence interval (vertical axis) of ${\overline{\epsilon }}$. (**a**) Result using probability density function $q_3(\cdot |u,v)$. (**b**) Result using probability density function $q_1(\cdot )$. For given values of the median on the horizontal axis, the points on the vertical axis indicate the values of the confidence interval (purple segment), arithmetic mean (orange), geometric mean (blue), and energy input rate (black). For readability, 300 points and 60 intervals are drawn out of 30,000 trials.

The result of the identical twin experiment using $q_3({\overline{\gamma }}-{\widehat{\gamma }}|{\widehat{\gamma }}-{\widetilde{\gamma }}, {\widehat{\gamma }}-\gamma ^{\sharp })$ is shown in Fig. 10a. In 28,497 trials out of 30,000 (about $95\%$), the true value of ${\overline{\gamma }}$ lies within the CI, which ensures the validity of the estimation method.

### Control experiments

To show the superiority of the proposed method (L3), which performs an inversion using the result of the joint PDF $q_3$ created by the cascade model with the Lévy generator, the generated samples above are also estimated by other methods: an inversion based on the marginal PDF $q_1$, an inversion based on the joint PDF generated by a multiplicative cascade with Gaussian generators, and a simple bootstrap method.

#### Error estimation based on the PDF of the arithmetic mean

Using several different moments for a profile should be effective for precise error estimation. To check this, we perform a control experiment using the marginal PDF, $q_1$, of the arithmetic mean (case L1). We take samples created by the cascade model with the stable Lévy generators, and then estimate the CI via the marginal PDF $q_1({\overline{\gamma }}-{\widehat{\gamma }})$. The results are shown in Fig. 10b, where the CI and median show a common positional relation to the arithmetic mean. Comparing the case with $q_3$ to the one with $q_1$, $75\%$ of trials in the former have narrower CIs. This indicates that using information from ${\widetilde{\gamma }}$ and $\gamma ^{\sharp }$ improves the error estimation.

#### Error estimation based on a cascade model with Gaussian generators

**Figure 11:**
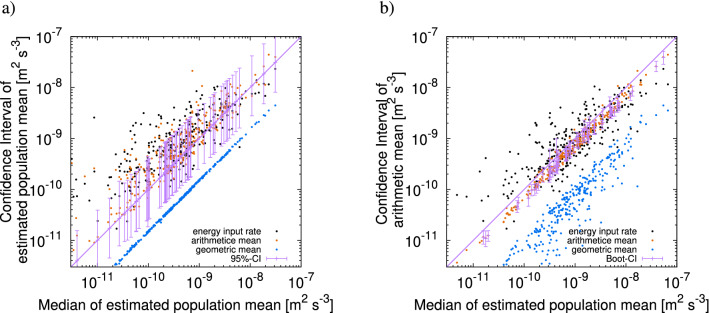
Result of control experiments. Median (horizontal axis) versus confidence interval (vertical axis) of ${\overline{\epsilon }}$, which are obtained using (**a**) joint probability density function based on the cascade model with Gaussian generators, and (**b**) the bootstrap method. For given values of the median on the horizontal axis, the points on the vertical axis indicate the values of the confidence interval (purple segment), arithmetic mean (orange), geometric mean (blue), and energy input rate (black). For readability, 300 points and 60 intervals are drawn out of 30,000 trials.

Stable Lévy generators have asymmetry in the distribution, which also affects the mean and median. Therefore, it is necessary to use such asymmetric generators for the error evaluation. To check this, we also evaluate the samples in the twin experiment using the probability distribution generated by a cascade model with Gaussian generators for comparison. We take samples created by the cascade model with stable Lévy generators (case G3) or Gaussian generators (case G1), and then estimate the CI via the joint PDF based on the cascade model with the best-fitted Gaussian generators. The results are shown in Fig. 11a, where a significant portion (9891 trials out of 30,000) of the true energy input rate (black) protrudes above the CI (purple). This means that the values of the energy input rate are underestimated if we assume a Gaussian distribution for the generators, and it also illustrates that it is inappropriate to use the statistics from the simulations of a cascade model with Gaussian generators for error evaluation.

#### Error estimation using the bootstrap method

The simplest method for error evaluation is applying the bootstrap method to each profile. However, the errors cannot be properly assessed by such a conventional method. To verify this, we estimate the CIs by applying the bootstrapping method to the twin experiment. For each trial, we use a 1000-member ensemble for the bootstrapping. Each member is constructed as follows: If a profile at the horizontal point $\vec {x}_i$ has a set of $J_i$ observations of the energy dissipation rate, $\epsilon _{r_0}(\vec {x},z^i_j),\quad j=1,2,\ldots ,J_i$, we randomly take $J_i$ samples with replacement from the set. The results of the error evaluation of the mean dissipation rate are shown in Fig. 11b. The CIs are evaluated very narrowly, and in many trials (22,164 trials out of 30,000), the true energy input rate (black) is outside the CI, which indicates that the error estimate is far too optimistic. This illustrates that it is irrelevant to use the conventional bootstrap method for the error evaluation.

#### Comparison of skill

For a fair comparison of skill in the above control experiments, we examine the errors for an estimator of the energy input rate. First, we define the estimator, $\Theta$, from the median estimate, $\gamma _{(0.5)}$, as

24 $$\begin{aligned} \Theta [m]\,&{\mathop {=}\limits ^{\text {def}}}\,a \exp {\left( \gamma _{(0.5)}[m]\right) }, \end{aligned}$$

25 $$\begin{aligned} \frac{1}{a}&= \frac{1}{M'} \sum _{m=1}^{M'} \frac{\exp {\left( \gamma _{(0.5)}[m]\right) }}{{\overline{\epsilon }}[m]} , \end{aligned}$$

where [*m*] represents the *m*-th sample, and $\gamma _{(0.5)}[m]$ is the median of the samples generated from the energy input rate ${\overline{\epsilon }}[m]$. Here, *a* is defined to empirically satisfy the unbiasedness:

26 $$\begin{aligned} \frac{1}{M'} \sum _{m=1}^{M'} \frac{\Theta [m]}{{\overline{\epsilon }}[m]}&=1. \end{aligned}$$

Then, in terms of $\Theta$, we define the relative error with sample size ${M'}$ as

27 $$\begin{aligned} \mathrm {ind}_{M'}&{\mathop {=}\limits ^{\text {def}}}\left( \frac{1}{M'} \sum _{m=1}^{M'} \left| \frac{{\overline{\epsilon }}[m]-\Theta [m]}{{\overline{\epsilon }}[m]}\right| ^2\right) ^{1/2}. \end{aligned}$$

Note that the error might not necessarily converge when ${M'}\rightarrow \infty$ because the distribution of $\Theta [m]/{\overline{\epsilon }}[m]$ is not Gaussian. Nevertheless, we can still evaluate the error for a finite ${M'}$ and use it for the comparison of skill.

The errors $\mathrm {ind}_{M'}$ with ${M'}=30{,}000$ for various conditions are listed in Table 3. The combinations of the cascade model with the Lévy generator or Gaussian generator and the use of joint PDF $q_3$ or marginal PDF $q_1$ are compared; these correspond to cases L3, L1, G3, and G1 above. The error for the arithmetic mean ${\widehat{\gamma }}$ is also shown for reference. The smallest error among these is for the estimator using the result of joint PDF $q_3$ created by the cascade model with a Lévy generator (L3). The errors for cases L1 and G1 are comparable to that for the arithmetic mean because these estimators are constructed from the statistics of the arithmetic mean. This result clearly shows that the proposed method (L3) can be used to define an estimator that yields superior estimates of the energy input rate than the arithmetic mean or other methods (L1, G3, or G1).

**Table 3:** Comparison of the error, $\mathrm {ind}_{M'}~({M'}=30{,}000)$, for various estimators in identical twin experiment.

$\mathrm {ind}_{M'}$	$q_3$		$q_1$	
Lévy	L3	0.89 (0.745)	L1	0.98 (0.718)
Gauss	G3	1.0 (1.61)	G1	0.98 (0.689)
${\widehat{\gamma }}$	0.99 (1.00)			

Lévy/Gaussian indicates the generator used in cascade model simulation. Here, $q_3$/$q_1$ indicates whether joint PDF or marginal PDF is used as the density generated by the cascade model simulation; ${\widehat{\gamma }}$ indicates the arithmetic mean. Each number in parentheses is the prefactor *a* for the corresponding estimator.

### Real data experiment

**Figure 12:**
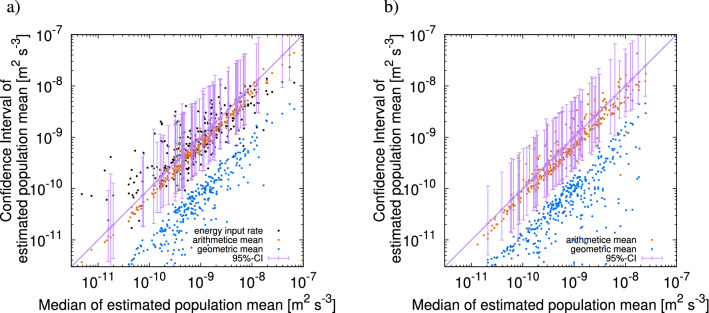
Results of the real data experiment compared with those of the identical twin experiment. Median (horizontal axis) versus confidence interval (vertical axis) of ${\overline{\epsilon }}$. (**a**) Results of identical twin experiment. (**b**) Results of real data experiment. For given values of the median on the horizontal axis, the points on the vertical axis indicate the values of the confidence interval (purple segment), arithmetic mean (orange), geometric mean (blue), and energy input rate (black). In b, only 70 confidence intervals out of 353 trials are shown for readability.

**Figure 13:**
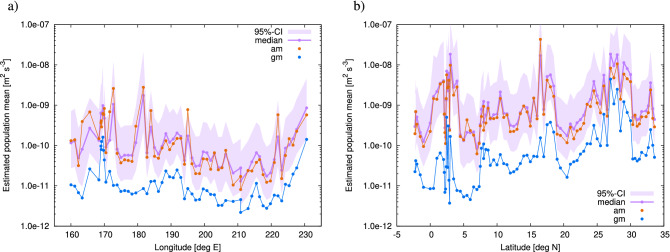
Geographical distribution of median (purple dots) and confidence interval (purple shade) of ${\overline{\epsilon }}$ along (**a**) $47^{\circ }~\mathrm {N}$ and (**b**) $137^{\circ }~\mathrm {E}$ . The horizontal axis shows the location. Arithmetic mean (orange) and geometric mean (blue) are also shown.

We applied the same procedure as in the identical twin experiment to the real observational profiles of the energy dissipation rate. Each profile was characterised by ${\widehat{\gamma }}$, ${\widehat{\gamma }}-{\widetilde{\gamma }}$, and ${\widehat{\gamma }}-\gamma ^{\sharp }$, which were utilised as observational constraints. By means of inversion, we derived the CI of ${\overline{\gamma }}$ for each profile at different horizontal locations. The estimated CIs for real data are shown in Fig. 12b; these CIs exhibit a similar appearance to the ones for the identical twin experiment in Fig. 12a. Figure 13 shows the estimate for CIs on the sections along $47^{\circ }~\mathrm {N}$ and $137^{\circ }~\mathrm {E}$. Along $47^{\circ }~\mathrm {N}$, the median of ${\overline{\epsilon }}$ rarely exceeds $10^{-9}~\mathrm{m}^2~\mathrm{s}^{-3}$, except around $172^{\circ }~\mathrm {E}$, $180^{\circ }~\mathrm {E}$, or $50^{\circ }~\mathrm {W}$. The peak of the arithmetic mean at $172^{\circ }~\mathrm {E}$ is approximately 2.5 times larger than the median estimate, which can lead to overestimation.

Along $137^{\circ }~\mathrm {E}$, the median shows several significant peaks over $10^{-8}~\mathrm{m}^2~\mathrm{s}^{-3}$ at around $2^{\circ }~\mathrm {N}$, $16^{\circ }~\mathrm {N}$, and 27 to $29 ^{\circ }~\mathrm {N}$. We could have underestimated the peaks at around $2^{\circ }~\mathrm {N}$ and 27–29$^{\circ }~\mathrm {N}$, but overestimated the one at around $16^{\circ }~\mathrm {N}$, if only the arithmetic means were used.

For the analysis of the observations on the section along $165^{\circ }~\mathrm {E}$, we should consider the effects of repeated observation. In fact, the observations were performed twice at some horizontal locations. For such cases, we simply assume that two independent realisations of a common ${\overline{\gamma }}$ are observed. In this regard, the inversion formula in Eq. (16) is modified as follows:

28 $$\begin{aligned} P({\overline{\gamma }}|{\widehat{\gamma }}_1,{\widehat{\gamma }}_2)&= \frac{P({\widehat{\gamma }}_1,{\widehat{\gamma }}_2|{\overline{\gamma }})P({\overline{\gamma }})}{\int P({\widehat{\gamma }}_1,{\widehat{\gamma }}_2|{\overline{\gamma }})P({\overline{\gamma }})\mathrm {d}{\overline{\gamma }}} = \frac{P({\widehat{\gamma }}_1|{\overline{\gamma }}) P({\widehat{\gamma }}_2|{\overline{\gamma }})P({\overline{\gamma }})}{\int P({\widehat{\gamma }}_1|{\overline{\gamma }}) P({\widehat{\gamma }}_2|{\overline{\gamma }}) P({\overline{\gamma }})\mathrm {d}{\overline{\gamma }}} = \frac{q_1({\overline{\gamma }}-{\widehat{\gamma }}_1) q_1({\overline{\gamma }}-{\widehat{\gamma }}_2)}{\int q_1({\overline{\gamma }}-{\widehat{\gamma }}_1) q_1({\overline{\gamma }}-{\widehat{\gamma }}_2) \mathrm {d}{\overline{\gamma }}}, \end{aligned}$$

where two observations are distinguished by the subscripts 1, 2. This distribution is the normalised product of the two distributions. When considering ${\widetilde{\gamma }}$ and $\gamma ^{\sharp }$, the same procedure as in Eq. (28) is applied to $q_3(\cdot |u,v)$ instead of $q_1(\cdot )$:

29 $$\begin{aligned}&P({\overline{\gamma }}|{\widehat{\gamma }}_1,{\widehat{\gamma }}_2, {\widehat{\gamma }}_1-{\widetilde{\gamma }}_1=u_1, {\widehat{\gamma }}_1-\gamma ^{\sharp }_1=v_1, {\widehat{\gamma }}_2-{\widetilde{\gamma }}_2=u_2, {\widehat{\gamma }}_2-\gamma ^{\sharp }_2=v_2 )\nonumber \\&\quad = \frac{q_3({\overline{\gamma }}-{\widehat{\gamma }}_1|u_1,v_1) q_3({\overline{\gamma }}-{\widehat{\gamma }}_2|u_2,v_2)}{\int q_3({\overline{\gamma }}-{\widehat{\gamma }}_1|u_1,v_1) q_3({\overline{\gamma }}-{\widehat{\gamma }}_2|u_2,v_2) \mathrm {d}{\overline{\gamma }}}. \end{aligned}$$

**Figure 14:**
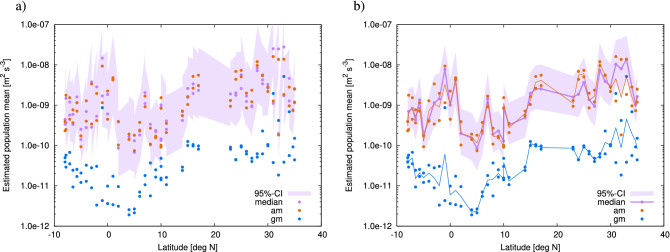
Geographical distribution of median (purple dots) and confidence interval (purple shade) of ${\overline{\epsilon }}$ along $165^{\circ }~\mathrm {E}$. The effect of repeated observation is considered in (**b**), but not in (**a**). The horizontal axis shows the location. Arithmetic mean (orange) and geometric mean (blue) are also shown.

Figure 14b shows the estimation of CI along $165^{\circ }~\mathrm {E}$ by taking into account the effect of repeated observation. For comparison, the result without considering the repeated observation is shown in Fig. 14a, where each observation is assumed to correspond to independent ${\overline{\gamma }}$. We can see that the CIs become narrower when considering the effect of repeated observation. Furthermore, along $165^{\circ }~\mathrm {E}$, the median shows a significant plateau on the order of $10^{-8}~\mathrm{m}^2~\mathrm{s}^{-3}$ at around $30^{\circ }~\mathrm {N}$, and a significant peak on the order of $10^{-8}~\mathrm{m}^2~\mathrm{s}^{-3}$ at around $2^{\circ }~\mathrm {S}$.

## Discussion

We have analysed the observed data obtained from oceanic turbulence measurements and shown that the vertical sequences of energy dissipation rates in a profile have an intermittent structure that obeys a scaling law. In this study, we have laid greater emphasis on the ’energy input rate’, which refers to the population mean for a profile, than on the ’mean energy dissipation rate’, which is the sample (arithmetic) mean over a profile. Based on the scaling property, we have proposed a method of estimating the energy input rate, given the sample statistics of an observed profile.

1. For scaling within the observed profiles, the statistical properties of our data are consistent with the universal multifractal model, which has a moment scaling exponent of $K(q)=\left( C_1/(\alpha -1)\right) \left( q^{\alpha }-q\right)$ with a multifractal index $\alpha = 1.62 \pm 0.03$ and codimension of the mean $C_1 = 0.352 \pm 0.009$. This result elucidates the universality that is inherent in the vertical structure of oceanic turbulence data.
2. The energy input rate and its uncertainty can be estimated using the results of Monte Carlo simulation of the cascade model with stable Lévy generators. This method computes the conditional probability, given the observed values of the arithmetic mean, geometric mean, and quadratic mean over a profile. The estimate provides additional information on the uncertainty of the energy input rate.
3. Furthermore, a comparison to control experiments has demonstrated that the proposed method is superior to a simple bootstrap method, an inversion based on the PDF generated by a multiplicative cascade with Gaussian generators, or an inversion based on the PDF of the arithmetic mean.
4. A real data experiment using the observed profiles has demonstrated a geographical distribution of the median estimates and confidence intervals of energy input rate, providing information on the range of values in which the turbulent energy can be dissipated per unit depth at each horizontal location.
5. Thus, we have found an answer to the question: ‘How can one estimate energy input rate from the vertical profile data of the energy dissipation rate?’ By analysing the intermittency in the observed data, we can construct a multiplicative cascade model based on the universal multifractal formalism that can reproduce the statistics of the data. Then, based on the observed data, the energy input rate can be estimated by inverting the probability distribution obtained from Monte Carlo simulations of the cascade model.
6. Since the observed sequence of the energy dissipation rate fluctuates greatly due to intermittency, it is difficult to extract robust information from an observed profile only by examining the average. Using the proposed method, it is possible to estimate the population mean for a profile even when repeated observations cannot be made at the same horizontal position. In other words, we can distinguish, to some extent, whether a profile shows an occasional large mean or whether the population mean itself is large. Therefore, more information can be extracted from a small amount of turbulence observation data, which can be a great advantage in regional data analysis.
7. Theoretically, this technique can easily be extended by utilising more statistics over a profile besides the arithmetic mean, geometric mean, or quadratic mean. Note however that such an extension may easily suffer from the curse of dimensionality, and thus, it can become impractical.
8. Even though we have used a discrete cascade model for simplicity and computational viability, we can extend it to a continuous cascade^33^, which may improve the estimation accuracy at the cost of increased computational burden.
9. To investigate the scaling of the velocity spectrum in the horizontal and vertical directions, we should apply anisotropic scaling theory (e.g., the Kolmogorov–Bolgiano–Obukhov model^23^). However, this study only aimed at investigating the scaling of the energy dissipation rate in the vertical direction in a purely statistical manner. Nevertheless, because the energy dissipation rate is one of the key quantities in scaling analysis, our results will contribute to further studies on how intermittency affects various scaling behaviours of turbulence in buoyancy-driven stratified fluids.

## Supplementary information

Supplementary Information.
