# Supplementary material for: Estimating the population mean for a vertical profile of energy dissipation rate
Source: Sci Rep. 2020 Nov 23;10:20414. doi: 10.1038/s41598-020-77414-2 (PMC7683546; doi:10.1038/s41598-020-77414-2)
Supplement: Supplementary file 1 — Supplementary Information. [file 41598_2020_77414_MOESM1_ESM.pdf]

Supplementary information for "Estimating the population mean for a vertical profile of energy dissipation rate"

Nozomi Sugiura[1,*], Shinya Kouketsu[1], Shuhei Masuda[1], Satoshi Osafune[1], and Ichiro Yasuda[2]

[1]Research and Development Center for Global Change, Japan Agency for Marine-Earth Science and Technology, Yokosuka, Japan
[2]Atmosphere and Ocean Research Institute, University of Tokyo, Chiba, Japan
[*]nsugiura@jamstec.go.jp

A Procedure for the estimation of rate ϵ_{r_0} from fast-response thermistor measurements

Microscale temperature fields were measured at 512Hz using the fast-response Fasttip Probe model 07 (FP07) thermistors. These were attached to the frames for measuring conductivity, temperature, and depth (CTD) as common oceanographic observational platforms. The turbulent energy dissipation rate was estimated from the spectrum of the temperature vertical gradient, $|\partial T'/\partial z|^2$. The estimation of ϵ_{r_0} is performed as follows.

1. From 512 data points of $\partial T'/\partial z$ on a $\sim$ 1m ($\sim$ 1s) vertical segment, an observation spectrum is drawn.

2. Using the method proposed in Goto et al. (2006)[5], the observation spectrum is corrected with a double-pole function with a time constant of 3ms. An example of observation spectrum is shown in Fig. A.1 by the blue curve.

3. For tracers in the fluid with the Prandtl number much larger than 1, which is the case with the seawater, the Kraichnan theoretical spectrum[7] is formulated as follows[9]:

$$S_{\text{theoretical}}(k; k_B) = \frac{\chi\sqrt{q_K}}{\kappa_T k_B} y_k^2 \frac{\exp\left(-\sqrt{6}y_k\right)}{y_k}, \tag{A.1}$$

$$y_k = \sqrt{q_K}\frac{k}{k_B}, \tag{A.2}$$

   where k is wavenumber; k_B is the Batchelor wavenumber; q_K is the Kraichnan constant, κ_T is the molecular thermal diffusivity, and χ is the thermal dissipation rate. q_K has been estimated as $q_K = 3.4 - 7.9$ (3.41: Antonia and Orlandi[1]; 5.26 ± 0.25: Bogucki et al.[3,4], 7.9 ± 2.5: Sanchez et al.[11]). We use a fixed value of $q_K = 5.26$, which was introduced in Bogucki et al.[3,4]. Note that this form of the spectrum is the modified version of the Batchelor spectrum[2] that considers the intermittency of strain fields. An example of a theoretical spectrum is shown in Fig. A.1 by the red curve.

4. The thermal dissipation rate, χ, in Eq. (A.1) is approximated as

$$\chi = 6\kappa_T \overline{\left(\frac{\partial T'}{\partial z}\right)^2}, \tag{A.3}$$

   where the overline denotes spatial averaging. This is estimated by numerically integrating the observational spectrum between the lowest wavenumber and the highest wavenumber at which the spectrum amplitude is more than 1.5 times the noise spectrum (light-blue curve in Fig. A.1).

5. After substituting the estimated value of χ, the theoretical curve (A.1) is fitted to the observed curve using the maximum likelihood method developed by Ruddick et al.[10] and by using the parameters proposed by Peterson and Fer[8].

6. By detecting the wavenumber at the spectrum peak:

$$k_P = \frac{k_B}{\sqrt{6q_K}}, \tag{A.4}$$

the Batchelor wavenumber, k_B, is determined.

7. Then, the energy dissipation rate, ϵ, is obtained using the following expression:

$$k_B = \frac{1}{2\pi}\left(\frac{\epsilon}{\nu\kappa_T^2}\right)^{\frac{1}{4}}. \tag{A.5}$$

8. Further, the ϵ data with large lowering speed variability (W_{sd}: standard deviation of the lowering speed in the 1s data) and slow lowering speed (W) were discarded with the criteria of $W_{\mathrm{sd}} > 0.2(W - 0.3)$ according to Goto et al.[6].

9. These quality controlled ϵ data were averaged in 10m-bins with half overlap, yielding ϵ_{r_0}.

Please refer to Goto et al. (2016, 2018)[5, 6] for more detailed estimation procedures.

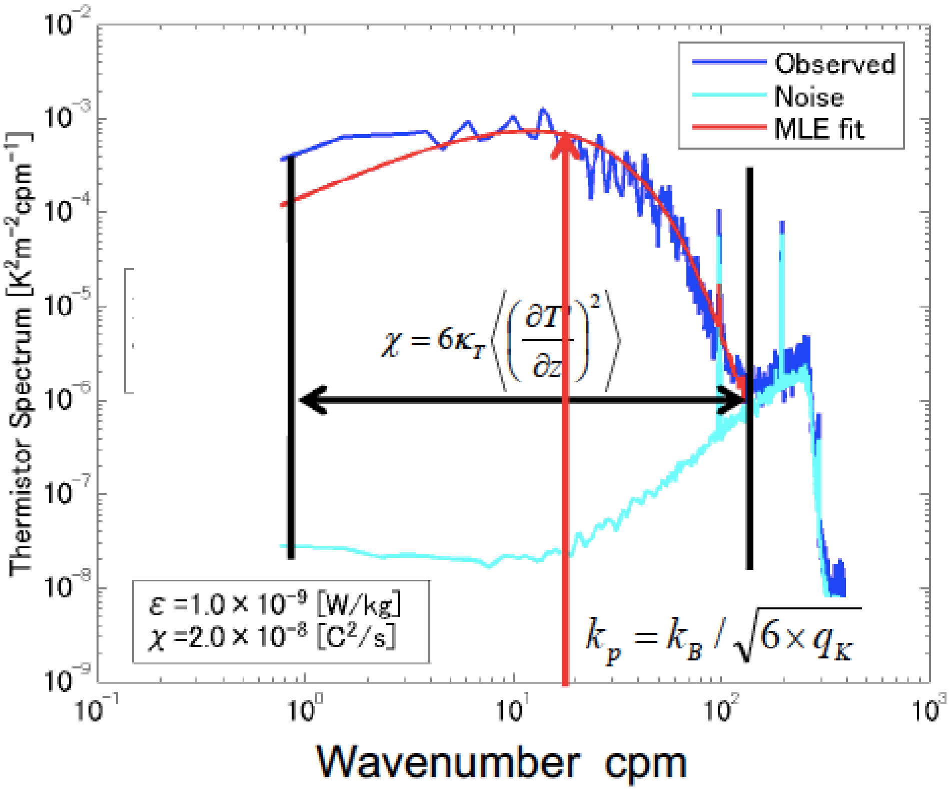

Figure A.1: Example of observational temperature gradient spectrum (blue curve), noise spectrum (light blue), and fitted theoretical spectrum (red) with the peak at k_P.

References

[1] RA Antonia and P Orlandi. On the Batchelor constant in decaying isotropic turbulence. *Physics of Fluids*, 15(7):2084–2086, 2003.

[2] G. K. Batchelor. Small-scale variation of convected quantities like temperature in turbulent fluid Part 1. General discussion and the case of small conductivity. *J. Fluid Mech.*, 5(1):113–133, 1959.

[3] Darek Bogucki, J Andrzej Domaradzki, and PK Yeung. Direct numerical simulations of passive scalars with $Pr > 1$ advected by turbulent flow. *Journal of Fluid Mechanics*, 343:111–130, 1997.

[4] DJ Bogucki, H Luo, and JA Domaradzki. Experimental evidence of the Kraichnan scalar spectrum at high reynolds numbers. *Journal of Physical Oceanography*, 42(10):1717–1728, 2012.

[5] Yasutaka Goto, Ichiro Yasuda, and Maki Nagasawa. Turbulence Estimation Using Fast-Response Thermistors Attached to a Free-Fall Vertical Microstructure Profiler. *Journal of Atmospheric and Oceanic Technology*, 33(10):2065–2078, 2016.

[6] Yasutaka Goto, Ichiro Yasuda, and Maki Nagasawa. Comparison of Turbulence Intensity from CTD-Attached and Free-Fall Microstructure Profilers. *Journal of Atmospheric and Oceanic Technology*, 35(1):147–162, 2018.

[7] Robert H Kraichnan. Small-scale structure of a scalar field convected by turbulence. *The Physics of Fluids*, 11(5):945–953, 1968.

[8] Algot K Peterson and Ilker Fer. Dissipation measurements using temperature microstructure from an underwater glider. *Methods in Oceanography*, 10:44–69, 2014.

[9] Elena Roget, Iossif Lozovatsky, Xavier Sanchez, and Manuel Figueroa. Microstructure measurements in natural waters: Methodology and applications. *Progress in Oceanography*, 70(2-4):126–148, 2006.

[10] Barry Ruddick, Ayal Anis, and Keith Thompson. Maximum Likelihood Spectral Fitting: The Batchelor Spectrum. *Journal of Atmospheric and Oceanic Technology*, 17(11):1541–1555, 2000.

[11] Xavier Sanchez, Elena Roget, Jesus Planella, and Francesc Forcat. Small-scale spectrum of a scalar field in water: the Batchelor and Kraichnan models. *Journal of Physical Oceanography*, 41(11):2155–2167, 2011.
